# Volumetric image interpretation in radiology: scroll behavior and cognitive processes

**DOI:** 10.1007/s10459-018-9828-z

**Published:** 2018-05-16

**Authors:** Larissa den Boer, Marieke F. van der Schaaf, Koen L. Vincken, Chris P. Mol, Bobby G. Stuijfzand, Anouk van der Gijp

**Affiliations:** 1 Utrecht University, Heidelberglaan 1, 3584 CS Utrecht, The Netherlands; 2 University Medical Center Utrecht, Utrecht, The Netherlands; 3 University of Bristol, Bristol, UK

**Keywords:** Radiology, Trainees, Volumetric image interpretation, Cognitive processes, Scroll behavior

## Abstract

The interpretation of medical images is a primary task for radiologists. Besides two-dimensional (2D) images, current imaging technologies allow for volumetric display of medical images. Whereas current radiology practice increasingly uses volumetric images, the majority of studies on medical image interpretation is conducted on 2D images. The current study aimed to gain deeper insight into the volumetric image interpretation process by examining this process in twenty radiology trainees who all completed four volumetric image cases. Two types of data were obtained concerning scroll behaviors and think-aloud data. Types of scroll behavior concerned oscillations, half runs, full runs, image manipulations, and interruptions. Think-aloud data were coded by a framework of knowledge and skills in radiology including three cognitive processes: perception, analysis, and synthesis. Relating scroll behavior to cognitive processes showed that oscillations and half runs coincided more often with analysis and synthesis than full runs, whereas full runs coincided more often with perception than oscillations and half runs. Interruptions were characterized by synthesis and image manipulations by perception. In addition, we investigated relations between cognitive processes and found an overall bottom-up way of reasoning with dynamic interactions between cognitive processes, especially between perception and analysis. In sum, our results highlight the dynamic interactions between these processes and the grounding of cognitive processes in scroll behavior. It suggests, that the types of scroll behavior are relevant to describe how radiologists interact with and manipulate volumetric images.

## Introduction

Radiology is a medical discipline in which images visualizing human bodies are examined for abnormalities. The interpretation of these images is considered a highly complex task since medical images are not self-explanatory (Drew et al. 2013a, b; Van der Gijp et al. 2014). Whereas the major part of current radiology practice applies volumetric, also known as three-dimensional (3D), imaging techniques, most research into the interpretation of radiological images is conducted on two-dimensional (2D) images. Research is needed because a deep understanding of volumetric image interpretation, involving the human–computer interactions such as scrolling, changing window settings, and viewing directions, is essential to improve training programs and feedback which are required to develop complex image interpretation skills (Nodine and Mello-Thomas 2010).

This study intends to contribute to the understanding of trainees’ volumetric image interpretation and to a theoretical framework of volumetric image interpretation in two ways. First, we focus on human–computer interactions in terms of scroll behavior as a fundamental aspect of volumetric image interpretation. We aim to distinguish different cognitive processes that are involved during different types of scroll behavior. In this way, we can reveal how scroll behavior and cognitive processes are related in volumetric image interpretation. Second, since little research studied relations between cognitive processes in volumetric image interpretation specifically, we aim to provide detailed insight into the relations between cognitive processes by focusing on transitions in cognitive processes.

### Perceptual and conceptual processing in 2D image interpretation

In most models of 2D image interpretation in radiology, perceptual and conceptual processing form the fundamental basis of the diagnostic process (Krupinski 2011; Kundel 2006). Whereas perceptual processing entails the identification of radiological findings in visual stimuli (Van der Gijp et al. 2014), conceptual processing encompasses understanding and attributing meaning to visual information, and is partly based on radiologists’ experience and knowledge of anatomy and pathology (Krupinski 2010a; Manning 2010). An example of an image interpretation model is the visual search and detection model of Kundel, Nodine, and Carmody (1978). They describe three main components in radiology image interpretation: (1) global impression; (2) focal search and attention; and (3) diagnostic decision making. This model suggests that the first glance at a medical image provides an overall, global orientation of the image. This global impression can be influenced by cognitive schemata and expectations. Subsequently, specific image parts that may have been detected in the impression phase are inspected, and are tested against schemata to reach a diagnosis in the end (Kundel et al. 1978; Kundel and Nodine 1983).

Besides the visual search and detection model, other image interpretation models exist. For example, the perceptual cycle theory which emphasizes the role of both cognitive schemata (Neisser 1976) and visual information in shaping actions and decisions, and the holistic perception model, which suggests that initial global image analysis produces a holistic perception that enables the rapid identification of abnormalities (Kundel et al. 2007). These models have in common that perceptual and conceptual processes are not considered isolated processes. Rather, they are regarded as interactive processes that together guide our attentional orienting. On the one hand, our attentional orienting can be under control of intentions, previous knowledge or expectations of the person who is attending (Theeuwes 2010). This is known as top-down processing. In medical image interpretation, top-down influences on attention may arise for example from patients’ medical history or radiologists’ previous experiences with the abnormality. On the other hand, attentional orientation can be influenced by stimulus characteristics, which is known as bottom-up processing (Itti and Koch 2001). For example, a large abnormality on a CT scan might stand out from the rest of the scan and therefore (automatically) attract attention.

Morita et al. (2008) studied the interactive relationship between perceptual and conceptual processing. They analyzed conceptual processing and external activities of five radiology experts and five radiology novices, which consisted of observing different types of computed tomography (CT) windows and writing reports, and found multiple influences of perceptual activity on the outputs of conceptual processing as well as influences of conceptual activity on the output of perceptual processing. More specifically, the interactions between perceptual and conceptual processing were most prominent in the experts in their study because they generated new perceptual features while writing the diagnostic report, a conceptual activity, and verbalized conceptual words while observing the images, a perceptual activity.

In addition to interactive relationships between perceptual and conceptual processes, research also suggests that it is sometimes hard to distinguish the two processes. This becomes apparent, for example, in eye-tracking studies revealing different causes of image interpretation errors (e.g., Drew et al. 2013a, b; Kundel et al. 1978). Errors can occur through failure to detect the abnormality, in which case eye-tracking shows no fixation on the abnormality at all (Kundel et al. 1978). Errors can also occur because of a failure to recognize or interpret the abnormality correctly, although the abnormality is fixated with the eyes (Krupinski 2010b; Kundel et al. 1978). The causes of the errors show an overlap of perceptual and conceptual processes.

### Influence of experience in image interpretation

Differences in type of case and radiologists’ level of experience can influence the interaction between perceptual and conceptual processing (Ericsson 2007; Kok et al. 2012; Lesgold et al. 1988; Morita et al. 2008). Both novices and experts change their viewing behavior according to different types of cases (Kok et al. 2012; Van der Gijp et al. 2017). It is also found that novices are often able to detect relevant information but that they cannot always interpret it correctly and integrate it into an accurate diagnosis, which points to interpretation errors (Kok et al. 2012). This suggests that novices’ image interpretation is largely driven by bottom-up processes (Morita et al. 2008). In contrast, experts’ viewing behavior is often influenced by top-down processes (Kok et al. 2012; Lesgold et al. 1988; Morita et al. 2008). This can be attributed to experts’ experience, larger knowledge base, and ability to ignore task-irrelevant information which are all likely to guide experts’ visual search (Lesgold et al. 1988).

### New framework for radiology image interpretation

**Fig. 1:**
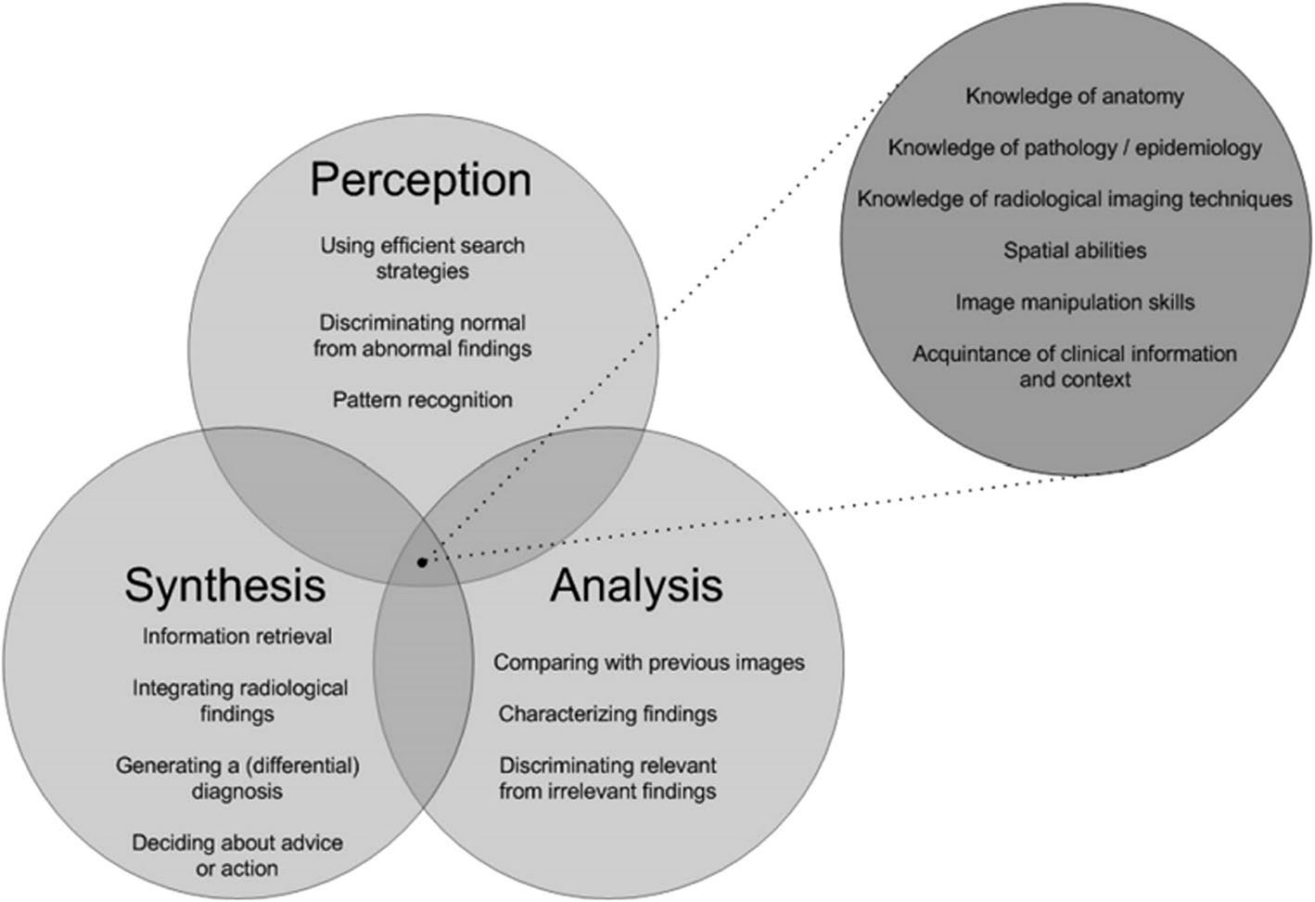
Framework of knowledge and skills in radiology image interpretation. From “Interpretation of radiological images: Towards a framework of knowledge and skills” by Van der Gijp et al. (2014), *Advances in Health Science Education.* Copyright (2014) by Advances of Health Education

The image interpretation models reviewed above describe general characteristics of processes during image interpretation. However, so far, specific knowledge and skills required for image interpretation are unclear or not described in these theories. In a study of Van der Gijp et al. (2014), a framework was developed that captures the knowledge and skills involved in radiology image interpretation (see Fig. 1). It contains three main components: perception, analysis, and synthesis. In this framework, perception entails for instance recognition of normal and abnormal findings, and analysis refers to characterizing findings and discriminating relevant from irrelevant findings. Synthesis encompasses, among others, the integration of findings and formulation of medical diagnosis and advice. The framework also has six requisite knowledge and skill items that can be related to each main component. The exact allocation of these items to one of the main components depends on the context.

In the current study, we use the terminology of the framework of Van der Gijp et al. (2014) to operationalize ‘cognitive processes’ because we consider a focus on specific skills and knowledge that emphasizes learning as more relevant for educational purposes than a focus on general characteristics of conceptual and perceptual processes (Van der Gijp et al. 2014). Furthermore, since the construction of the framework is based on an extensive literature research in combination with expert consultations and a verbal protocol experiment, we consider this model as a valid model to operationalize cognitive processing.

### Volumetric image interpretation

Models on image interpretation processes of 2D images may not be generalized to the volumetric image interpretation processes because of fundamental differences between the two imaging techniques (Van der Gijp et al. 2016). Volumetric images are composed of a set of ‘slices’ that are presented in a ‘stack’. A slice is a single 2D image of a cross section of the human body. Multiple slices together form a stack of images, that is, the volumetric image, also known as multi-sliced images. A fundamental difference in volumetric image interpretation compared to 2D image interpretation is that volumetric image interpretation involves more comprehensive human–computer interactions. When reading volumetric images, radiologists need to view and scroll through a substantial number of image slices. Besides scrolling, radiologists need to manipulate (e.g., changes in viewing directions and/or window settings) the image in such a manner that abnormalities become visible. Due to scrolling and other image manipulations, the visual information changes continuously (Van der Gijp et al. 2016). This information needs to be ‘fused’ into a single mental representation of the entire volumetric anatomic region being imaged (Krupinski 2010a). In terms of the framework of Van der Gijp et al. (2014), it is also found that trainees’ cognitive processes differ substantially during volumetric and 2D image interpretation: perception was more prominent in volumetric image interpretation, while synthesis was highly predominant in 2D image interpretation (Van der Gijp et al. 2015).Therefore, the interpretation of volumetric images is considered a more complex and time-consuming process than 2D image interpretation (Al-boukai et al. 2011; Husmann et al. 2007).

Research into volumetric imaging has been conducted in context of model-observers (e.g., Gifford 2012), which are mathematical models or computer algorithms that can evaluate the performance of volumetric imaging methods on the detection and characterization of abnormalities. Research into volumetric imaging addressing the relation between human visual search (strategies), perception, cognition and performance in volumetric image interpretation is, however, limited, as indicated by a review of Venjakob and Mello-Thoms (2015). Some researchers investigated how to apply eye tracking to volumetric images to describe gaze behaviors during interpretation of volumetric images in metrics (e.g., Helbren et al. 2014). Drew et al. (2013a, b), for example, focused on visual strategies in terms of eye movements and distinguished between two systematic visual strategies: observers who went through the volumetric image slice by slice, and scanned the whole slice before going to the next one (scanners) and observers who kept their eyes relatively still, limiting their search to a single quadrant of the anatomical region (drillers). Other researchers investigated the influence of expertise on visual search of volumetric images. For example, Cooper et al. (2009) examined observer performance in CT and MR stroke images, and found among other things that experts showed more fixations and were faster in detecting the primary lesions, and that novices made more false positive decisions. The researchers suggested that these differences in visual scanning of CT images may be due to experts’ complex cognitive maps with many preconceptions about anatomy, disease development and identification of abnormalities (e.g., top-down processing).

As touched upon before, scroll movements constitute an essential human–computer interactivity in volumetric image interpretation because they change the visual information. A study of Venjakob et al. (2012) focused on these scroll movements and suggest that three different scroll paths can be distinguished. These movements, or parameters can be calculated from the scroll path through the slices. First, ‘full runs’ are movements back and forward through more than 50% of the slices. Second, ‘half runs’ concern movements back and forward through 25 up to 50% of the slices. Third, ‘oscillations’ represent back and forward movements through 1 up to 25% of the slices. This study of Venjakob et al. (2012) showed that scroll movements resemble eye movements (despite a gap in performance across participants); longer time to first fixation and initial dwell times were associated with more runs and oscillations.

### The current study

Image interpretation models developed for 2D images may not be applicable to volumetric images since interpreting a stack of images differs fundamentally from searching a single 2D image (Krupinski 2010a; Van der Gijp et al. 2016). Therefore, the aim of the current study is to gain insight into trainees’ volumetric image interpretation in two ways. The first part of the current study focuses on human–computer interactions in terms of scroll behavior. More specifically, we examine how trainees’ cognitive processes differ during different types of scroll behavior. The cognitive processes, categorized into perception, analysis, and synthesis according to the framework of Van der Gijp et al. (2014), are investigated with think-aloud protocols, which is a frequently used method to identify complex reasoning mechanisms (Ericsson 2007). Regarding scroll behavior, we identify five types of which the first three types are derived from the study of Venjakob et al. (2012) and concern full runs, half runs, and oscillations. Since runs are thought to reflect global search (Venjakob et al. 2012), we expect full runs and half runs to be stronger related to perception than oscillations. Oscillations are considered scroll movements during which specific structures are systematically compared (Venjakob et al. 2012). We therefore expect oscillations to be stronger related to analysis and synthesis than full runs and half runs. Since it is also possible to change viewing direction (e.g., from axial to coronal viewing direction) and window setting (e.g., from bone to tissue setting) in volumetric images, we include these behaviors which we refer to as image manipulations. Finally, we also look at interruptions, which are moments at which the scrolling is intermitted. We investigate the cognitive processes during image manipulations and interruptions in an exploratory way.

The second part of the study focuses on the relations between perception, analysis, and synthesis. As outlined in the introduction, so far most research is conducted on 2D images and focuses on perceptual and conceptual processes that are thought to be interactively related (e.g., Kundel et al. 1978; Morita et al. 2008). Furthermore, it is suggested that trainees’ image processing is largely driven by bottom-up processing (Kok et al. 2012; Lesgold et al. 1988; Morita et al. 2008). By operationalizing cognitive processing in terms of perception, analysis, and synthesis (Van der Gijp et al. 2014), this study can shed a different light on the relation between perceptual and conceptual processing since we use different and more concrete terminology concerning skills and knowledge used in radiology. To understand the relations between cognitive processes during volumetric image interpretation, we focus on transitions by investigating which cognitive processes are likely to follow each other during trainees’ volumetric image interpretation process. Although the types of cognition are probably alternating throughout the image interpretation process, we expect that in general perception is followed by analysis, and analysis, in turn, is followed by synthesis. This would reflect a bottom-up process.

## Methods

### Participants

Data of the research project of Van der Gijp et al. (2015) are used in the current study. The participants in this dataset are 20 fourth to sixth-year radiology trainees (M_age_ = 34.7 years, 75% women) of the University Medical Center Utrecht (UMCU) in the Netherlands. The project of Van der Gijp et al. (2015) focused on this intermediate-level study population because intermediates are likely to verbalize more than both novices and experienced radiologists do (Rikers et al. 2000). All participants agreed to volunteer in this study and signed informed consent forms.

### Instrumentation

#### Image cases

Participants were asked to complete four CT volumetric image cases, selected from a larger set of 17 possible CT image cases. Image cases were read in stack mode implying that several hundreds of image slices were stacked up in order to reconstruct volumetric human anatomical structures. All 17 cases involved prevalent (sub-)acute diseases in four radiology subareas; neuroradiology (four cases of traumatic brain diseases or strokes), musculoskeletal (four cases of fractures of shoulder, hip, foot, or spine), abdominal (five cases of acute diseases of abdominal organs, vessels or bowel, such as traumatic, vascular or infectious problems), and chest (four cases of acute diseases of the lungs or vessels, such as traumatic, vascular or infectious problems). Each participant made one case out of each subarea. This was in line with the objectives of the traineeship the participants were undertaking at the time, which was completed by all participants.

A short clinical vignette with information about patients’ age, gender, relevant history and complaints, was presented during the introduction of each image case. It is found that clinical backgrounds influence radiologists’ performance as well as their viewing behavior (Berbaum et al. 2010). However, since radiologists usually view a medical image in combination with a clinical background, we argue that this reflects a natural situation of image interpretation and improves ecological validity. Moreover, providing a clinical background corresponds with the objectives of the traineeship.

#### VQuest

The image cases were shown in VQuest (http://www.vquest.nl), a digital assessment environment that allows scrolling through a stack of images, changing viewing direction or window settings, zooming in and out, and panning the image. VQuest is used for radiology assessments at UMCU as well as at a national level and is found to be valid, accurate, and user-friendly (Ravesloot et al. 2015).

#### Image display

**Fig. 2:**
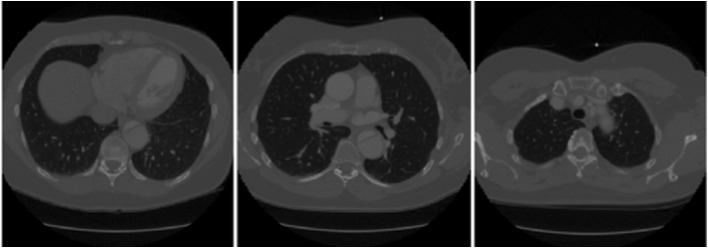
Example of image display of abdominals in axial viewing direction

The volumetric CT images cases were displayed in gray scale and were presented on a 23-inch computer monitor with a 1920 × 1080 pixel resolution (no calibration to DICOM GSDF). Participants were able to scroll back and forth through the stack of images. Besides scrolling through the image, window settings could be adjusted in soft tissue, bone, lung, and brain setting. Moreover, images cases could all be viewed in sagittal, coronal, and axial viewing direction (x, y, z). Figure 2 shows an example of a volumetric image case with three different image slices in the axial viewing direction of an image case.

### Procedure

The study took place in a computer room at the UMCU. Window blinds in this room were closed, blocking out most of the day light but not creating a totally dark environment, which is in accordance with daily radiology practice. Prior to the investigation, participants received a standard instruction and completed an exercise in thinking out loud. Subsequently, participants were asked to think aloud while examining the image cases. Participants were also asked to report a (differential) diagnosis, give advice if necessary, and to indicate whether they felt the need for additional information to interpret the image case. Participants were able to type this information into VQuest. A supervisor stayed in the room to encourage participants to think aloud. A video camera recorded the full procedure.

#### Performance

Participants received a score of 0 when the case was incorrectly solved, a score of 0.5 when the case was partly correctly solved (e.g., when the correct abnormality was reported but a mistake was made in the exact location of the abnormality, or when it was correctly reported that the abnormality was an aortic dissection but the incorrect type of dissection was mentioned), or a score of 1 when the case was completely correctly solved.

#### Coding think-aloud protocols

After transcription of the think-aloud protocols, the verbalizations were coded using the framework of Van der Gijp et al. (2014) as coding scheme (Table 2 in Appendix). This framework proved to have high interrater reliability in a former study (Cohen’s *κ* = .83) (Van der Gijp et al. 2014). Two independent raters coded the concurrent think-aloud protocols of 25% of the participants. After interrater reliability was found to be satisfactory (Cohen’s *κ* = .76), one rater continued to code the remaining think aloud protocols.

Utterances related to requisite knowledge and skills (see the requisite component in Fig. 1) could all be assigned to the main categories of perception, analysis, and synthesis based on the context of the specific utterance. By way of illustration, utterances reflecting manipulations of the image (e.g., “Let’s go to bone setting”, translated from Dutch) could be coded as perception in case a participant was searching for abnormalities, but a similar utterance could also be coded as analysis in case the participant manipulated the image with the purpose to analyze an abnormality observed previously. Utterances which could not be assigned to one of the main categories or to the requisite knowledge and skills items were categorized as ‘other’.

#### Identification types of scroll behavior

The types of scroll behavior were calculated from the logfiles, which were continuously recorded by VQuest. Logfiles kept track of the exact slice number that was depicted in the viewing direction (axial, sagittal, or coronal) at any moment. All manipulations were logged which resulted in 1500 to over 6000 recordings per image case.

**Fig. 3:**
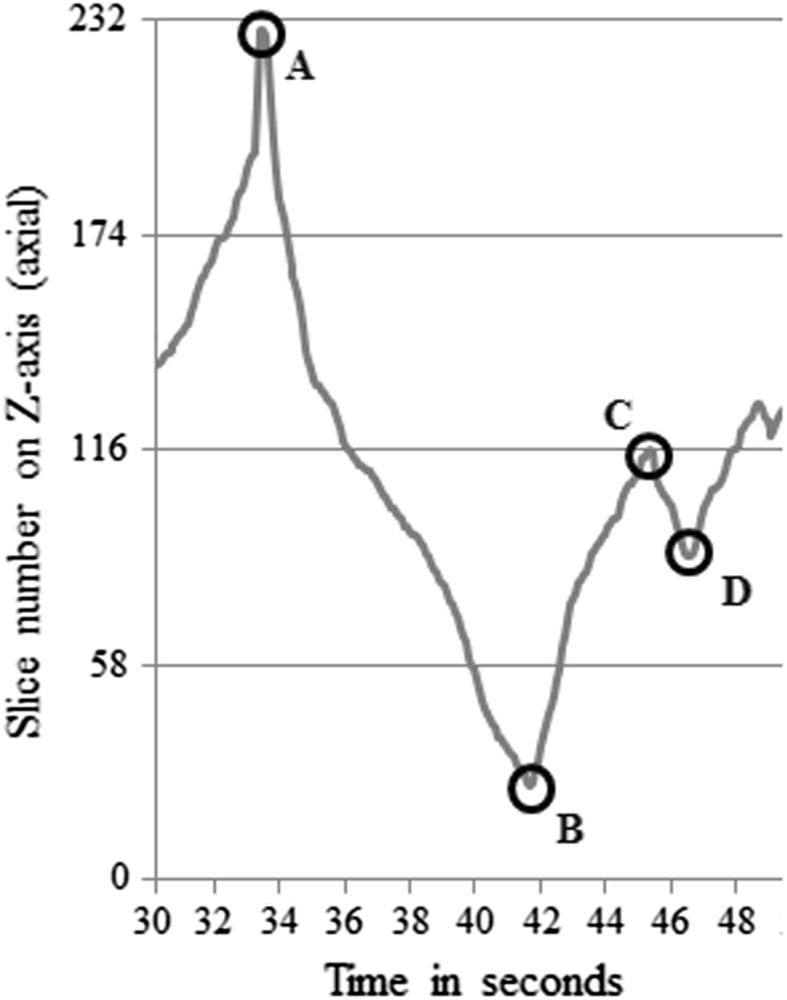
Graphical representation of a random part of the logfile of an image case viewed in axial direction

We identified full runs, half runs, or oscillations according to the definitions of Venjakob et al. (2012). Figure 3 shows a random part of a logfile on the Z-axis, that is, axial viewing direction, generated from an image case completed by a participant. Each time a local extreme minimum was reached, we compared this point to the previous local extreme maximum (and vice versa: extreme local maxima were compared to previous extreme local minima). In Fig. 3, letter B denotes an extreme local minimum, and A is its previous extreme local maximum. The difference between these two points on the Z-axis is more than 50% of the total number of slices in the current viewing direction, which was 232 in this case. Therefore, the A–B line shows a full run. The B–C line shows a half run, a scroll movements forward through 25–50% of all image slices. An oscillation is represented by the C–D line because this line represents a movement backward through less than 25% of all image slices. We treated scrolling movements through less than 1% of the slices as random noise and hence ignored these movements. In each image case, the total number of slices in each viewing direction differed (sagittal, coronal, and axial). The calculations of full runs, half runs, and oscillations were adapted to the total number of slices when the participant changed the viewing direction.

With respect to the two additional types of scroll behavior we introduced (i.e., image manipulations and interruptions) calculations were made as follows. Gaps in the logfiles, or in other words, an absence of logfile records for three or more seconds (not displayed in Fig. 3) were referred to as interruptions. Interruptions could occur during full runs, half runs, and oscillations. Regarding image manipulations, these behaviors were explicitly indicated in the logfiles by statements such as ‘changed to coronal’ indicating a change of viewing direction, or ‘changed to brain setting’ indicating a change of window settings. The entire process of extracting the types of scroll behavior from logfiles was performed in ImageXplorer (version 1.2.0.0), a program for log analyses, and was fully automatic.

### Data analysis

One case of one participant was not fully recorded and therefore deleted from the dataset. Eventually, the 20 participants completed 79 cases in total.

#### Performance

We checked whether the percentages of participants’ cognitive processes (i.e., perception, analysis, and synthesis) were related to performance on case and participant level. We calculated (1) Spearman’s correlation coefficients between case scores and the percentages of the three cognitive processes for that case and (2) Pearson’s correlation coefficients between participants’ total performance and their average percentage of the three cognitive processes, which could identify relations on participant level. Participants’ total performance was the sum of participants’ case scores and had a range of 0 (i.e., no correctly completed cases) to 4 (i.e., all cases correctly completed) with intervals of 0.5. Mean imputation of performance was applied for missing cases.

#### Scroll behavior and cognitive processes

To analyze whether and how types of scroll behavior and cognitive processes are related, we connected these two categorical variables based on time. Each time a participant verbalized a thought, a participant also executed a certain type of scroll behavior. Subsequently, we cross-tabulated the frequencies of the two categorical variables and performed a Chi-square test. Subsequent pairwise comparisons based on *z*-tests (resulting in z-statistics, the standard normal deviate) were calculated to compare the proportions of cognitive processes during full runs, half runs, and oscillations. All pairwise comparisons were adjusted with the Bonferroni correction in order to reduce the risk of making Type I errors.

Assumptions of the cross-tabulation analysis with Chi-square test were met. Although participants completed four cases each, independency of observations is assumed because previous research indicated that search behavior depends to a great extent on the case (Kok et al. 2012; Van der Gijp et al. 2017). In that sense, we assume that the cases made by one participant do not affect each other in terms of scroll behavior and cognitive processes.

#### Relations between cognitive processes

In order to analyze the relations between perception, analysis, and synthesis, we first described time patterns in cognitive processing throughout the image interpretation process. In line with Morita et al. (2008), we divided each image case into four equal phases of 25% of the total time because each case was completed in a different time length. For each phase, the percentages of utterances of perception, analysis, synthesis, and other were calculated over all participants and image cases. In the study of Morita et al. (2008), verbalizations representing conceptual and perceptual processing were also investigated throughout time and showed clear differences in verbalizations across the four phases. Therefore, we assumed that four equal phases are valid to identify transitions in cognitive processing through time.

Besides a time pattern description, we performed a lag sequential analysis to gain further insight into the relations between perception, analysis, and synthesis in terms of transitions. The data system of the think-aloud procedure allowed continuous sequential recording of cognitive processes, which resulted in a ‘stream’ of cognitive processes. Lag sequential analysis implies that we examined the conditional probability that a specific cognitive process (the ‘given’ code) is followed by another cognitive process (the ‘target’ code) (cf. Chorney et al. 2010). For example, we examined the conditional probability that perception is followed by analysis. Conditional probabilities were calculated as the probability of event X given event Y, according to the following formula: p(X|Y) = p(X, Y)/p(Y).

Since the lag sequential analysis concerns a stream of cognitive processes, we added two extra ‘events’ to identify the moments at which a switch was made between two cases and the moments at which the clinical information was read which was provided as part of the image cases. In this way, calculations of transitions were started over again at the beginning of a new image case, and thus, prevented the lag sequential analysis from miscalculations. The lag sequential analysis was performed in Multiple Episode Protocol Analysis (MEPA, version 4.9), which is a program for the coding and analysis of (non-)verbal observational data and protocols.

## Results

The average case score was 0.68 (*SD* = 0.35). The average total performance score of participants was 2.71 (*SD* = 1.07). We checked for relations between performance and cognitive processes. On case level, we found a positive relationship between the performance on a case and the percentage of synthesis on that case (*r*_*s*_ = .30, *p* < .001), indicating that the higher the performance on a case, the higher the percentage of synthesis. No relation was found between the performance and the percentages of perception (*r*_*s*_= − .13, *p* = .27) or analysis (*r*_*s*_ = − .06, *p* = .63) on case level. On participant level, we found no correlations between participants’ total performance score and participants’ average percentages of perception (*r*_*s*_= − .06, *p* = .80), analysis (*r*_*s*_= − .30, *p* = .21), or synthesis (*r*_*s*_= .37, *p* = .11). This means that participants’ total performance was not related to their average percentages of cognitive processes.

### Scroll behavior and cognitive processes

**Fig. 4:**
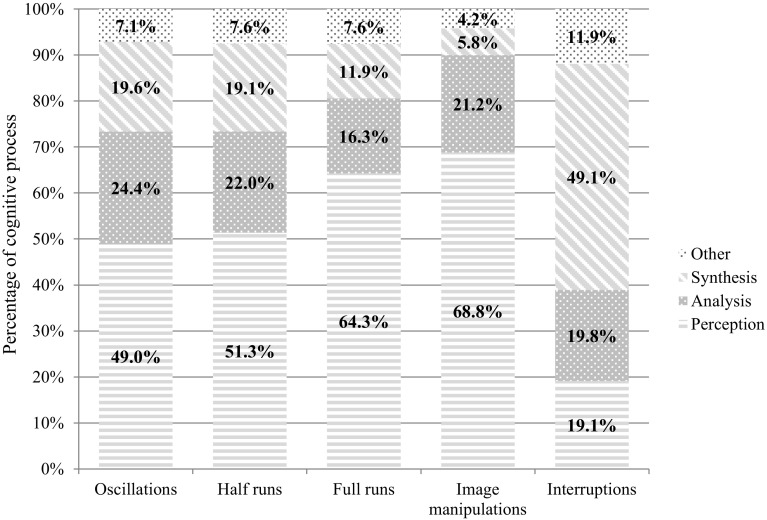
Cognitive processes during different types of scroll behavior

In total, 5590 segments in think-aloud protocols, divided over the 79 completed image cases, were categorized into utterances of perception, analysis, synthesis, and other. On average, this equals 70.76 think-aloud segments per participant, per image case (*SD *= 44.43). In total, 5482 think-aloud segments (98.1%) could be matched to a type of scroll behavior based on time. One hundred eight segments could not be matched because these utterances were made before the image case was displayed, so no logfiles were recorded yet. The overall Chi-square of the cross-tabulation analysis, as visualized in Fig. 4, was χ^2^(12) = 759.52, *p* < .001, indicating that type of scroll behavior and cognitive processes were dependent.

With respect to oscillations, half runs, and full runs, the pairwise comparisons (with a Bonferroni adjustment resulting in an adjusted alpha of .017) between these three types of scroll behavior provided additional information. First, although oscillations, half runs, and full runs all coincided with many utterances of perception (49.0, 51.3 and 64.3% respectively), full runs coincided with significantly more utterances of perception than oscillations, *z *= 7.52, *p *< .001, and half runs, *z *= 5.70, *p *< .001. Second, the percentages of utterances of analysis and synthesis during oscillations and half runs, as shown in Fig. 4, were significantly higher than the percentage of utterances of analysis and synthesis during full runs (analysis: *z*_*oscillations vs full runs*_= 4.79, *p *< .001; *z*_*half runs vs full runs*_= 3.17, *p *< .001, synthesis: *z*_*oscillations vs full runs*_= 4.97, *p *< .001; *z*_*half runs vs full runs*_= 4.31, *p *< .001). Third, oscillations and half runs did not differ significantly from each in terms of utterances of perception, *z* = 1.27, *p *= .20, analysis, *z* = 1.48, *p *= .14, and synthesis, *z* = 0.29, *p *= .76. In sum, the results indicate a distinction between cognitive processes during full runs on the one hand, and oscillations and half runs on the other hand.

We also proposed two additional types of scroll behavior, namely interruptions and image manipulations. Figure 4 indicates that utterances of perception accounted for the major part in image manipulations. Interruptions, on the contrary, mostly consisted of utterances of synthesis. The percentage of utterances of analysis and perception during interruptions are both lower than the percentage of utterances of synthesis.

### Relations between cognitive processes

**Fig. 5:**
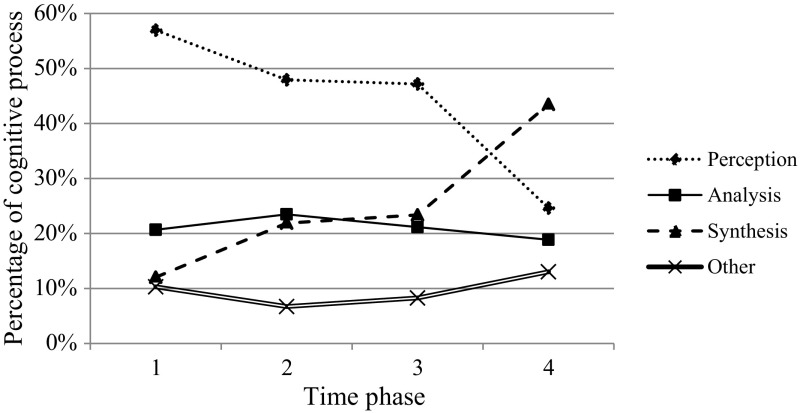
Descriptive time patterns in cognitions. Perception: T1 > T2/T3 > T4. Analysis: T1/T3 < T2 < T4. Synthesis: T1 < T2/T3 < T4

The descriptive time patterns of cognitive processes are displayed in Fig. 5 showing that all cognitive processes occurred in each of the four phases, though in different proportions. More specifically, perception was predominantly present in the first three time phases. In the fourth time phase, synthesis became dominant. Analysis never played a dominant role compared to perception and synthesis. Analysis increased from the first to the second phase but decreased after the second phase.

We further analyzed the transitions in cognitive processes in a lag sequential analysis. In total, 2618 transition segments from one cognitive process or events into different cognitive processes or events were registered, including the task switches and reading clinical information. On average, each participant made 32.89 transitions per case (*SD* = 19.85). In comparison with an average of 70.76 segments per image case, it can be suggested that 46.5% of the next segments in the ‘stream’ involved a different cognitive process.

Table 1 demonstrates quantitative results of the lag sequential analysis and gives the conditional probabilities of transitions in cognitive processes during the interpretation of volumetric images. It can be derived that participants started a new image case always with reading the clinical information. After reading the clinical information, participants were most likely to verbalize in the category of perception. Moreover, before completing the task, most utterances were labeled as synthesis.

**Table 1:** Conditional probabilities and absolute frequencies in brackets of sequences in trainees’ cognitive processes during volumetric image interpretation

Given code	Target code						
	Perception	Analysis	Synthesis	Other	Clinical information	Case switch	Total
Perception	–	47.2% (390)*	32.9% (272)*	18.9% (156)	–	< 1.0% (8)	100% (826)
Analysis	49.5% (312)*	–	36.7% (231)*	13.7% (86)	–	< 1.0% (1)	100% (630)
Synthesis	40.6% (254)*	31.0% (194)*	–	20.5% (128)*	–	8.0% (50)*	100% (626)
Other	51.2% (194)*	12.1% (46)*	31.4% (119)*	–	–	5.3% (20)*	100% (379)
Clinical information	83.5% (66)*	–	5.1% (4)	11.4% (9)	–	–	100% (79)
Case switch	–	–	–	–	100% (78)*	–	100% (78)
Total	31.6% (826)	24.1% (630)	23.9% (626)	14.5% (379)	3.0% (78)	3.02% (79)	100% (2618)

Percentages are based on totals of the rows

*z > 1.96, *p* < .05

Furthermore, Table 1 contains information about the relation between perception, analysis, and synthesis. The *z*-scores of the conditional probabilities between perception, analysis, and synthesis are significant, which indicates that transitions between perception, analysis, and synthesis were more likely to occur than may be expected based on the null hypothesis (i.e., a proportional distribution of all target codes). More specifically, Table 1 shows that perception is more often followed by analysis than by synthesis, and a pairwise comparison adjusted with Bonferroni correction indicates that the probability that analysis follows perception is also significantly higher than the probability that synthesis follows perception, *z* = 5.92, *p *< .001. Furthermore, the probability that analysis is followed by perception is higher than the probability that analysis is followed by synthesis, *z* = 4.61, *p *< .001. This indicates that especially close interactions between perception and analysis exist. Synthesis is most often followed by perception, *z* = 3.54, *p *< .001. However, there is no difference in whether analysis or perception precede synthesis, *z* = 1.49, *p *= .13.

## Discussion

Although volumetric imaging is daily practice in radiology, there is little understanding of the volumetric image interpretation process since prior research into the understanding of the image interpretation process in radiology has been conducted on 2D images (e.g., Kok et al. 2012; Kundel and Nodine 1983; Lesgold et al. 1988). However, models on 2D image interpretation may not be applicable to volumetric image interpretation because of fundamental differences between 2D and volumetric images. In the current study, we aimed to gain insight into the volumetric image interpretation process as experienced by radiology trainees.

The first part of the current study focused on human–computer interactions in terms of scroll behavior as a fundamental aspect of volumetric image interpretation and showed that cognitive processes differ during different types of scroll behavior. Our results confirm the hypothesis that full runs, which were thought to be more reflective of global search than oscillations (Venjakob et al. 2012) coincide more often with cognitive processes of perception than oscillations do. This means that global characteristics of, and abnormalities in the medical image are largely detected during full runs. The results regarding oscillations are also in line with our hypothesis stating that systematic comparison of maximally a fourth of the slices coincides more often with cognitive processes of analysis and synthesis than scrolling through a very large set of slices at once. Our findings corroborate those of Venjakob et al. (2012) by showing that oscillations might be more reflective of in-depth processing compared to full runs. Surprisingly, the proportions of cognitive processes during half runs were almost identical to oscillations instead of to full runs, that is, less perception and more analysis and synthesis than during full runs. Half runs, like oscillations, may therefore also be more reflective of in-depth processing. Our results also indicate that combining full runs and half runs into one category of ‘runs’, as was done in Venjakob et al. (2012), might not be valid as they imply different cognitive processes.

Interestingly, during oscillations and half runs, the majority of cognitive processing concerned perception. This might be explained by general features of volumetric images. Volumetric image interpretation requires more perceptual processing since volumetric images contain more visual information (Husmann et al. 2007; Krupinski 2010a; van der Gijp et al. 2015). To locate abnormalities, perceptual processes may therefore also involve comparing specific slides, and this behavior would be characterized by oscillations and half runs.

With respect to the two additional types of scroll behavior, we found that image manipulations were to a great extent characterized by utterances of perception, indicating that images were generally manipulated in order to search for abnormalities in the image, rather than for analyzing or synthesizing abnormalities. Furthermore, we found that interruptions mainly coincided with synthesis, meaning that participants were mainly integrating findings and generating medical diagnoses and advice during interruptions. A plausible explanation is that during interruptions, participants were frequently typing the diagnosis and advice into the software which was part of the task. It makes sense that during writing the medical diagnosis and advice, many utterances of synthesis are made because this task requires the integration of information. In the study of Morita et al. (2008), it was also found that when participants wrote reports, radiologists summarized features that had already been found in earlier stages, and made diagnostic decisions. In the second part of the study, we focused on the relation between perception, analysis, and synthesis by investigating transitions between these cognitive processes. With respect to the time patterns in cognitive processes, we found that after reading the clinical information, perception was dominant until the third phase and that synthesis substantially increases in the last time phase. The fairly dominant role of perception at the start of the image interpretation process may also point to perceptual orientation on and global impression of the image case in the beginning (Kundel et al. 1978). These results reflect an overall bottom-up way of reasoning in trainees’ volumetric image interpretation process, implying that throughout the time of reading an image case, information is integrated into larger components and eventually integrated into a final decision about medical diagnosis and advice (Manning 2010). A bottom-up way of reasoning supports previous research into image interpretation in trainees (Lesgold et al. 1988; Morita et al. 2008; Kok et al. 2012).

The lag-sequential analysis revealed that within this global bottom-up way of reasoning, many interactions between perception, analysis, and synthesis existed, which is comparable to the ideas of close interactive relations between perceptual and conceptual processing (e.g., Morita et al. 2008). In line with our expectations, we found that perception is more often followed by analysis than by synthesis. Contrary to our expectations, analysis is more likely to be followed by perception than by synthesis. It might therefore be suggested that especially perception and analysis are closely and interactively related.

Since we operationalized cognitive processing by the framework of Van der Gijp et al. (2014), this study is able to shed a new and more concrete light on the content of the proposed relations between perceptual and conceptual processing, and on the specific content of bottom-up processing. We found that perception and analysis were closely related to each other and that analysis was never the dominant cognitive process throughout the image interpretation process. It can be suggested that especially analysis is supportive of perception and that the product of the interactions between perception and analysis lead to synthesis. Thus, whereas ‘bottom’ (as in bottom-up) is largely explained by perceptual verbalizations (e.g., Morita et al. 2008) or visual fixations in eye-tracking (e.g., Kok et al. 2012) in previous research, our study suggests that ‘bottom’ might already imply interactions between especially perception and analysis. In other words, it means that the process of attending to and recognizing abnormalities, and the analysis of these abnormalities are interwoven. Moreover, since our results indicated that synthesis was closer related to perception than to analysis, it can be suggested that what might be referred to as ‘up’ (as in bottom-up) also implies interactions between synthesis and perception and, to a lesser extent, between synthesis and analysis.

Importantly, rather than treating the categories of the framework of Van der Gijp et al. (2014) as a one-way information process from perception, to analysis, to synthesis, our study suggests that cognitive processes should be seen as interactive components. Furthermore, the cognitive processes are dynamic since they were constantly changing throughout the image interpretation process. These dynamic interactions between the cognitive processes can be placed within dynamic system theories on cognition, which highlight that cognitive processes are complex, non-linear, and dynamic (Fusella 2013). Although we cannot make direct comparisons with interactions between cognitive processes in 2D images, the features of volumetric images are likely to reinforce the dynamic interactions between cognitive processes because human–computer interactions cause a continuous change of visual information (Van der Gijp et al. 2016). New visual information requires new perceptual search to and analysis of (ab)normal structures which need to be fused into one mental representation (Krupinski 2010a). The suggestion that dynamic interactions in cognitions are stimulated by new visual information is supported by research outside radiology. Visual design research for example, highlights the occurrence of dynamical relations between perception and conceptual ideas due to discoveries of new visual information in sketches (Suwa et al. 2000).

With respect to the human–computer activities, which cause this continuous change of visual information, our study may also fit with ideas of the embodiment of cognition in dynamic system theories by showing that cognitive processes differ during different types of scroll behavior. The relationship between cognitive processes and scroll behavior indicates that in volumetric image interpretation, action and cognitive processes are not separate modules. Rather, cognitive processes are grounded in perceptual and motoric systems (Barsalou 2008), meaning that executed actions fulfill functional roles in cognitive processes of perception, analysis and synthesis (cf. De Koning, and Tabbers 2011).

Our results should be discussed in the light of some limitations. First, whereas the current study focused on describing general patterns in scroll behaviors and cognitive processes and therefore used a wide range of cases, a note of caution should be raised regarding the influence of case types on cognitive processes. The correlation analyses between performance and cognitive processes showed that on case level, more synthesis was related to higher performance on a case. However, on participant level, a higher average percentage of synthesis was not related to a higher total performance. This was also the case for perception and analysis. On the one hand, this might indicate that participant level differences (such as total performance or ability) may have influenced the percentages of cognitive processes to a limited extend. On the other hand, other factors on case level might have affected participants’ percentages of the cognitive processes. Cognitive processes may, in other words, be more dependent on case specific characteristics than on participant characteristics. This is in line with research that suggests that observers’ viewing behavior changes according to the type of case (Kok et al. 2012; Van der Gijp et al. 2017). Therefore, it is important for further research to examine the influence of case level differences on patterns of cognitive processes and examine into what extend the general patterns found in our study can be found in specific cases. Second, the validity of the think-aloud methodology used in our study depends on the number of reformulations and on the time interval between the occurrence of a thought and its verbal report (Ericsson 2006). Especially in our study, in which we matched verbalized cognitive processes and scroll behavior based on time, misinterpretations of relations between the two variables were at risk if, for example, a verbalization was reported later than it occurred in the mind. In such a case, that verbalization might have been incorrectly related to another type of scroll movement. Despite these this concern, we regard the think-aloud procedure as one of the only methods to capture complex reasoning processes and we tried to minimize this time interval risk by training the participants in thinking out loud.

A third limitation of the current study is that the 20 participants were trainees, and thus, all had the same experience as radiologist (intermediate). This means caution must be applied, as the findings of the current study are not transferable to radiologists with other levels of experience. Differences between radiologists can be expected since it is generally recognized that perceptual and bottom-up processes play important roles in novices’ image interpretation, as was found in this study as well, whereas more experienced radiologists display more top-down processes (Lesgold et al. 1988; Kok et al. 2012).

Research into volumetric image interpretation so far has focused on how to the development of model-observers (e.g., Gifford 2012) or on relations between human visual search (with eye tracking), perception, cognition and performance (e.g., Drew et al. 2013a, b; Helbren et al. 2014). Our study adds the potential for developing guidelines and perspectives of the characterization of volumetric image interpretation by showing that scroll behaviors may also give relevant insight into the human volumetric image interpretation process. Using a large amount of logfile and think-aloud data which was recorded for 20 participants for the entire duration of the image interpretation process, we were able to further validate full runs, half runs, and oscillations, interruptions, and image manipulations as relevant parameters to describe and understand (patterns in) scroll behavior in future research into volumetric image interpretation. We also showed that scroll behavior in volumetric image interpretation may be no random actions. Rather, each type of scroll behavior appears to allow for different cognitive processes. Furthermore, since our study identified dynamic interactions in cognitive processing as a basic underlying component in volumetric image interpretation, another implication is that radiology education should acknowledge these dynamic interactions between cognitive processes by enabling students to arrange cognitive processes in a flexible manner. To inform radiology education about best practices, future studies should identify which patterns in cognitive processes including their dynamic interactions, and which patterns in scroll behavior are related to higher diagnostic accuracy.

## Appendix

See Table 2.

**Table 2:** Coding framework for think-aloud protocols

Category	Code
*Perception*	
Knowledge about anatomy	1.1
Knowledge of radiological imaging techniques	1.2
Knowledge about pathology/epidemiology	1.3
Using efficient search strategies	1.4
Image manipulations (scrolling, viewing directions, window settings)	1.5
Discriminating normal from abnormal findings	1.6
Pattern recognition	1.7
*Analysis*	
Knowledge about anatomy	2.1
Knowledge of radiological imaging techniques	2.2
Knowledge about pathology/epidemiology	2.3
Characterizing findings	2.4
Image manipulations (scrolling, viewing directions, window settings)	2.5
Distinguishing relevant from irrelevant findings	2.6
Comparing with previous images of the patient	2.7
*Synthesis*	
Knowledge about anatomy	3.1
Knowledge of radiological imaging techniques	3.2
Knowledge about pathology/epidemiology	3.3
Integrating radiological findings	3.4
Image manipulations (scrolling, viewing directions, window settings)	3.5
Generating a (differential) diagnosis	3.6
Deciding about advice or action	3.7
Information retrieval	3.8
Other	4

Adapted from: “Interpretation of radiological images: towards a framework of knowledge and skills” by Van der Gijp et al. (2014), *Advances in Health Science Education.* Copyright (2014) by Advances of Health Education
